# Ventricular Tachycardia as a Manifestation of Cardiac Metastasis Mimicking Acute Coronary Syndrome: A Case Report

**DOI:** 10.7759/cureus.89744

**Published:** 2025-08-10

**Authors:** Mohamed Mounir Nesnassi, Inasse Bargach, Rajae Zidouh, Hamza Chraibi, Lilian Marty, Kevin Sanchis, Rabeh Ghenim, Marc Andrieu, Taha Hassani

**Affiliations:** 1 Cardiology, Hôpital Jacques Puel, Centre Hospitalier de Rodez, Rodez, FRA

**Keywords:** cardiac mass, cardiac metastasis, extrinsic compression of coronary artery, minoca, ventricular tachycardia

## Abstract

We report the case of a 64-year-old male patient with a history of heart failure with midrange ejection fraction (EF), confirmed by cardiac MRI, and a coronary angiography performed three years earlier showing only mild coronary lesions. His past medical history also included a pulmonary embolism and lingual squamous cell carcinoma treated with radiotherapy, which remained in remission for three years.

He presented to the emergency department with syncope, which was found to be secondary to ventricular tachycardia with a left bundle branch block morphology and a negative QRS morphology in the inferior leads, as well as in lead I and aVL, consistent with a midseptal origin of the tachycardia. Intravenous amiodarone was administered, successfully restoring sinus rhythm. Transthoracic echocardiography revealed no significant structural abnormalities aside from a midrange EF. On the following day, coronary angiography revealed a long 60% stenotic lesion in the mid left anterior descending artery (LAD), which could represent either an atherosclerotic lesion or a parietal hematoma. A similar irregular lesion was observed in the first diagonal branch. A cardiac MRI was performed and revealed a septal myocardial mass with radiological features suggestive of a secondary (metastatic) lesion, potentially explaining the arrhythmic event and the stenotic lesion. A whole-body CT scan showed multiple secondary lesions in the liver, kidneys, adrenal glands, peritoneum, gluteal muscle, and lungs, as well as a pulmonary embolism. Unfortunately, the patient died one month after discharge.

This case highlights how a cardiac mass, likely metastatic, may present with ventricular arrhythmias and mimic an acute coronary syndrome, with a coronary angiography that can also be misleading. It also underscores the pivotal role of cardiac MRI in establishing the correct diagnosis when echocardiography and angiography yield inconclusive or atypical findings.

## Introduction

Cardiac metastases are an under-recognized but clinically significant manifestation of advanced malignancy. While most commonly arising from lung, breast, or hematologic cancers, metastases from head and neck tumors, particularly oral squamous cell carcinoma, are exceedingly rare [1]. These secondary cardiac lesions can present with a broad spectrum of nonspecific cardiac symptoms, ranging from chest pain to arrhythmias, and may mimic primary cardiac or coronary pathologies [2,3]. Ventricular arrhythmias, although uncommon, can be the initial clinical manifestation, leading to a diagnostic challenge, especially in patients with concurrent cardiovascular risk factors or preexisting heart disease [4]. Electrocardiographic changes and modest elevations in troponin levels are common but non-specific, and may mimic acute coronary syndromes [2,5]. Advanced imaging modalities such as cardiac MRI and CT are essential for diagnosis, as they allow better tissue characterization and identification of metastatic lesions [1].

We present the case of a 64-year-old male patient with a history of oral cavity carcinoma in remission admitted for ventricular tachycardia secondary to cardiac metastasis and mimicking an acute coronary syndrome. This case illustrates the diagnostic challenge of cardiac metastasis mimicking an acute coronary syndrome and highlights the importance of cardiac imaging in cancer patients presenting with arrhythmias. It also emphasizes the possible mechanism of coronary artery compression by the cardiac tumor, mimicking other causes of acute coronary syndromes, particularly parietal hematoma.

## Case presentation

We report the case of a 64-year-old male patient with a medical history of heart failure with midrange ejection fraction (EF) at 45% confirmed by cardiac MRI, and a coronary angiography performed three years prior, showing only mild coronary lesions. He also had a Holter ECG showing non-sustained ventricular tachycardia. His past history also includes a pulmonary embolism and lingual squamous cell carcinoma treated with radiotherapy, which remained in remission for three years. His treatment includes methotrexate 2.5 mg per day, which was continued after the cancer treatment, bisoprolol 5 mg per day, and empagliflozin 10 mg per day.

**Figure 1:**
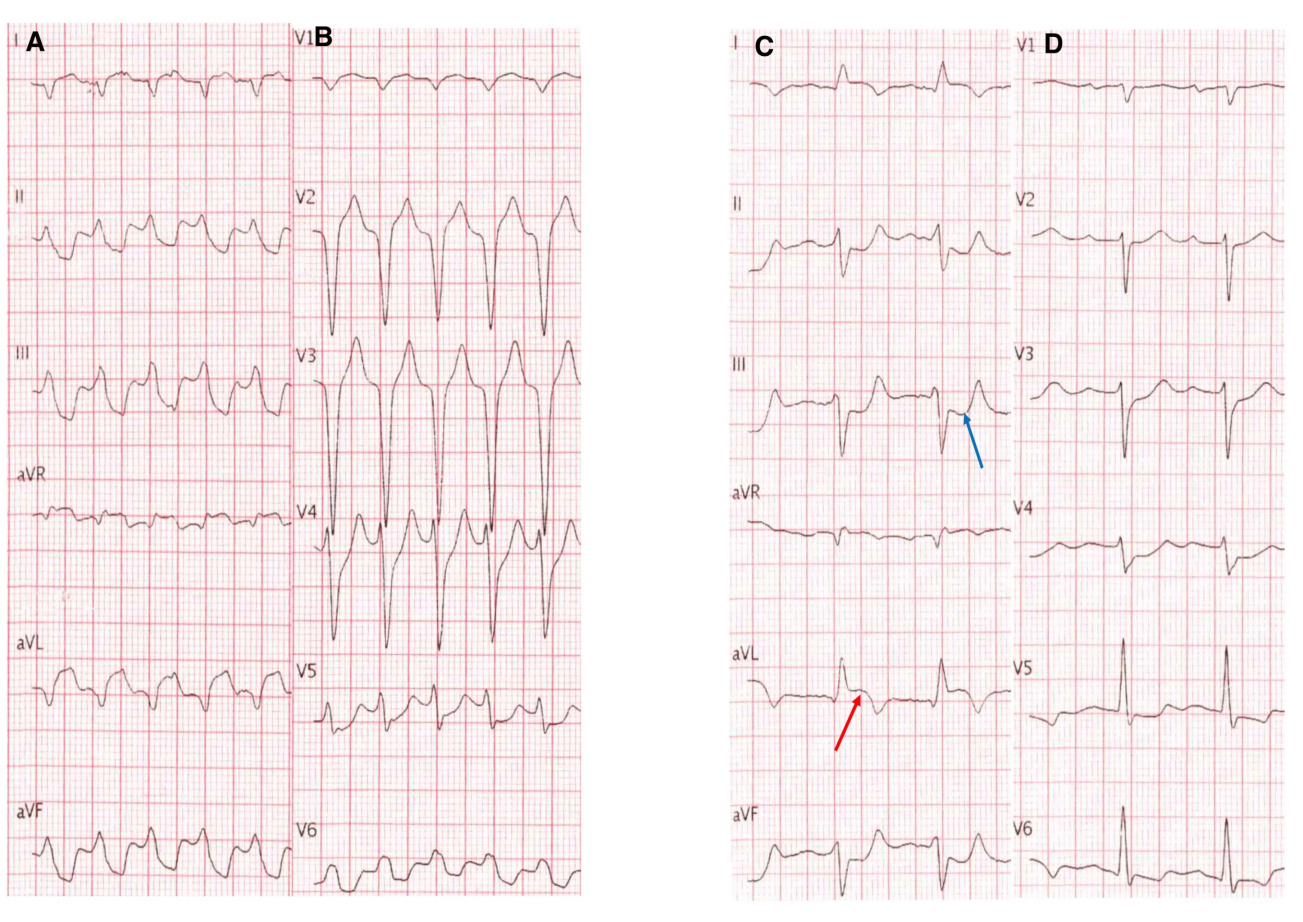
ECG of the patient A+B: ECG on admission showing ventricular tachycardia at 163 bpm with a left bundle branch block morphology and a negative QRS morphology in the inferior leads as well as in lead I and aVL consistent with a midseptal origin of the tachycardia. C+D: Post-electrical cardioversion showing sinus rhythm with a PR interval at 233 ms, with repolarization abnormalities: ST depression in inferior and anterior leads (blue arrow) and ST elevation in lead I and aVL with negative T waves in the same leads (red arrow)

The patient was admitted to the emergency department following a syncope. Electrocardiogram (ECG) showed a ventricular tachycardia at 163 beats per min (bpm) with a left bundle branch block morphology and a negative QRS morphology in the inferior leads, as well as in lead I and aVL, consistent with a midseptal origin of the tachycardia. Sinus rhythm was restored with intravenous amiodarone. ECG at sinus rhythm showed persistent repolarization abnormalities with ST depression in inferior and anterior leads and ST elevation in lead I and aVL with negative T-waves in the same leads (Figure 1).

**Figure 2:**
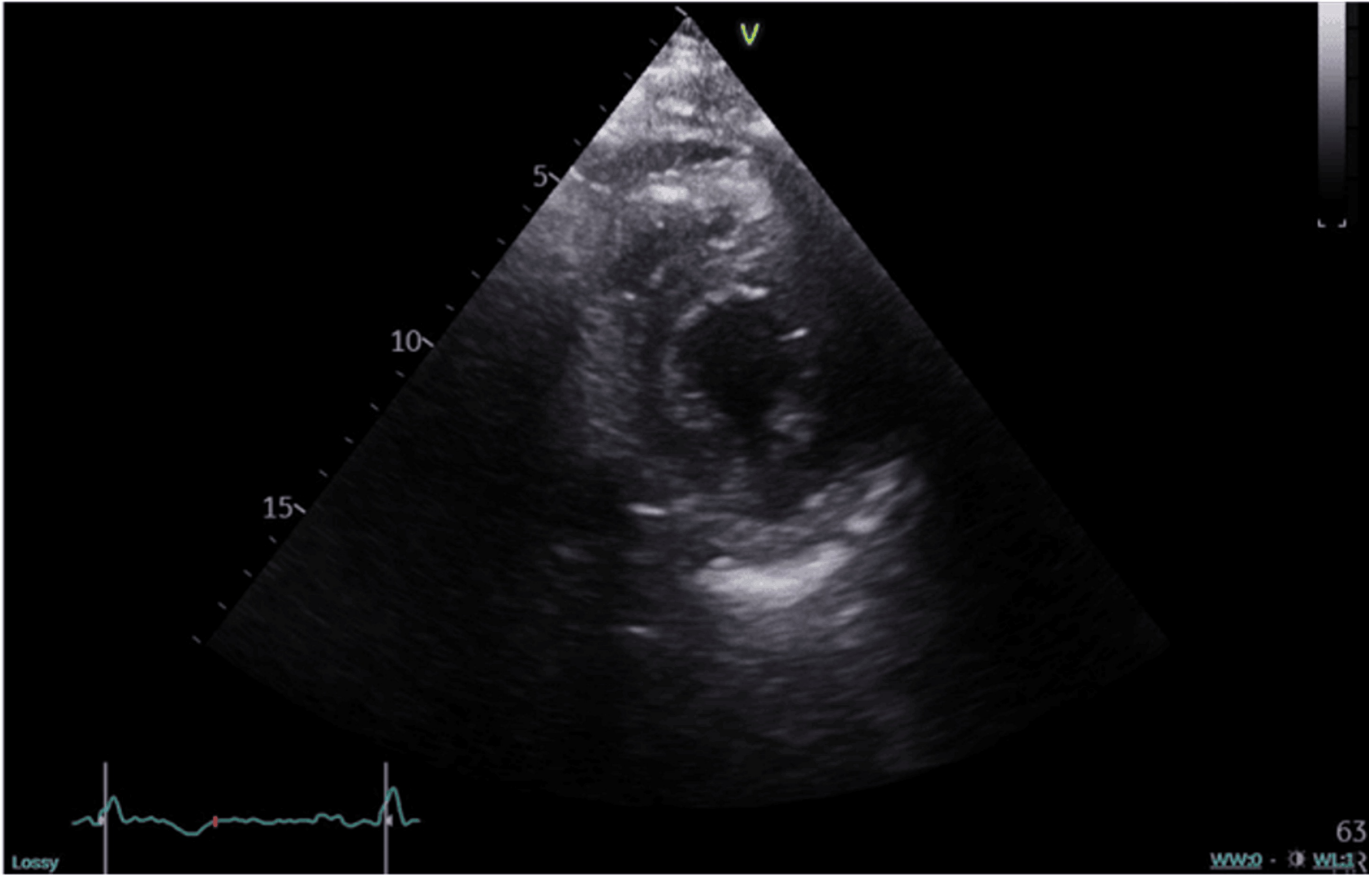
Parasternal short axis view from echocardiogram In this parasternal short axis view from echocardiogram, the cardiac structures are normal. The cardiac mass is not seen.

Transthoracic echocardiography showed normal cardiac chamber dimensions with left ventricular EF at 45%. The rest of the echocardiogram was normal (Figure 2).

Laboratory tests showed a troponin level at 170 ng/L, then decreasing to 70 ng/L the next day (normal range <14 ng/L, which represents the upper limit of the 99th percentile, with a strong probability of myocardial injury if higher than 52 ng/L). Other laboratory tests, such as complete blood count, potassium level, and renal function, were in the normal range (Table 1).

**Table 1:** Venous blood test NT-proBNP: N-terminal pro-B-type natriuretic peptide

Venous blood test	Patient value	Normal range
Potassium	4.02 mmol/L	3.4-4.5 mmol/L
Creatinine	8 mg/L	7-12 mg/L
Calcium	113 mg/L	88-102 mg/L
NT-proBNP	8910 pg/mL	< 900 pg/mL for acute heart failure; < 125 pg/mL for chronic heart failure
Troponin	Day 1: 170 ng/L; Day 2: 70 ng/L	< 14 ng/L (upper limit of the 99th percentile); strong probability of myocardial injury if higher than 52 ng/L or delta > 5 ng/L

**Figure 3:**
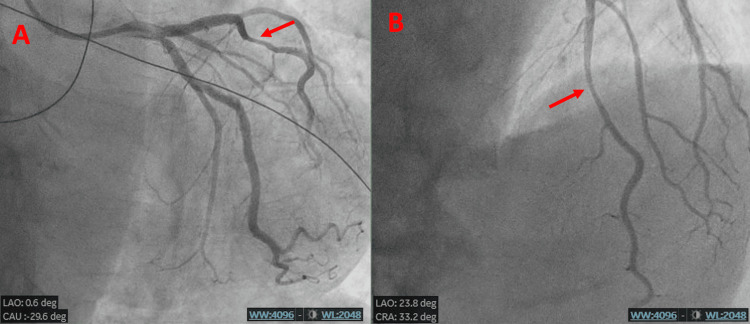
Coronary angiography images showing the suspect lesion in the mid LAD artery (red arrow) A: Caudal view. B: Cranial with left anterior oblique view. We can see the lesion in both views. It is a long 60% stenotic plaque in the mid left anterior descending (LAD) artery, appearing to be coronary hematoma.

Coronary angiography performed the next day revealed a long 60% stenotic plaque in the mid left anterior descending (LAD) artery, possibly corresponding to a mural hematoma (Figure 3). A similar irregular long plaque was noted in the first diagonal branch.

**Figure 4:**
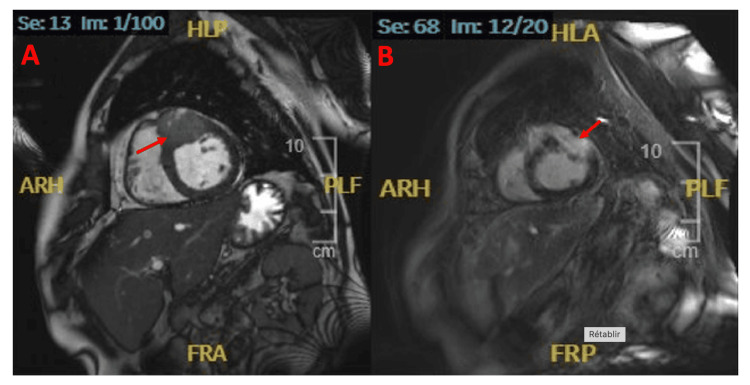
Cardiac MRI images showing the cardiac metastatic mass (red arrows) A: Short axis distal view. B: Short axis distal view with late gadolinium enhancement. Both views show the cardiac mass (measuring 25X20 mm involving the myocardium and located in the mid anteroseptal region) that was not seen in the short axis view from echocardiogram.

To further evaluate the findings, a cardiac magnetic resonance imaging (MRI) was performed five days after admission, revealing a myocardial mass measuring 25x20 mm involving the myocardium and located in the mid anteroseptal region, related to the course of the narrowed artery. The mass demonstrated late gadolinium enhancement (Figure 4).

**Figure 5:**
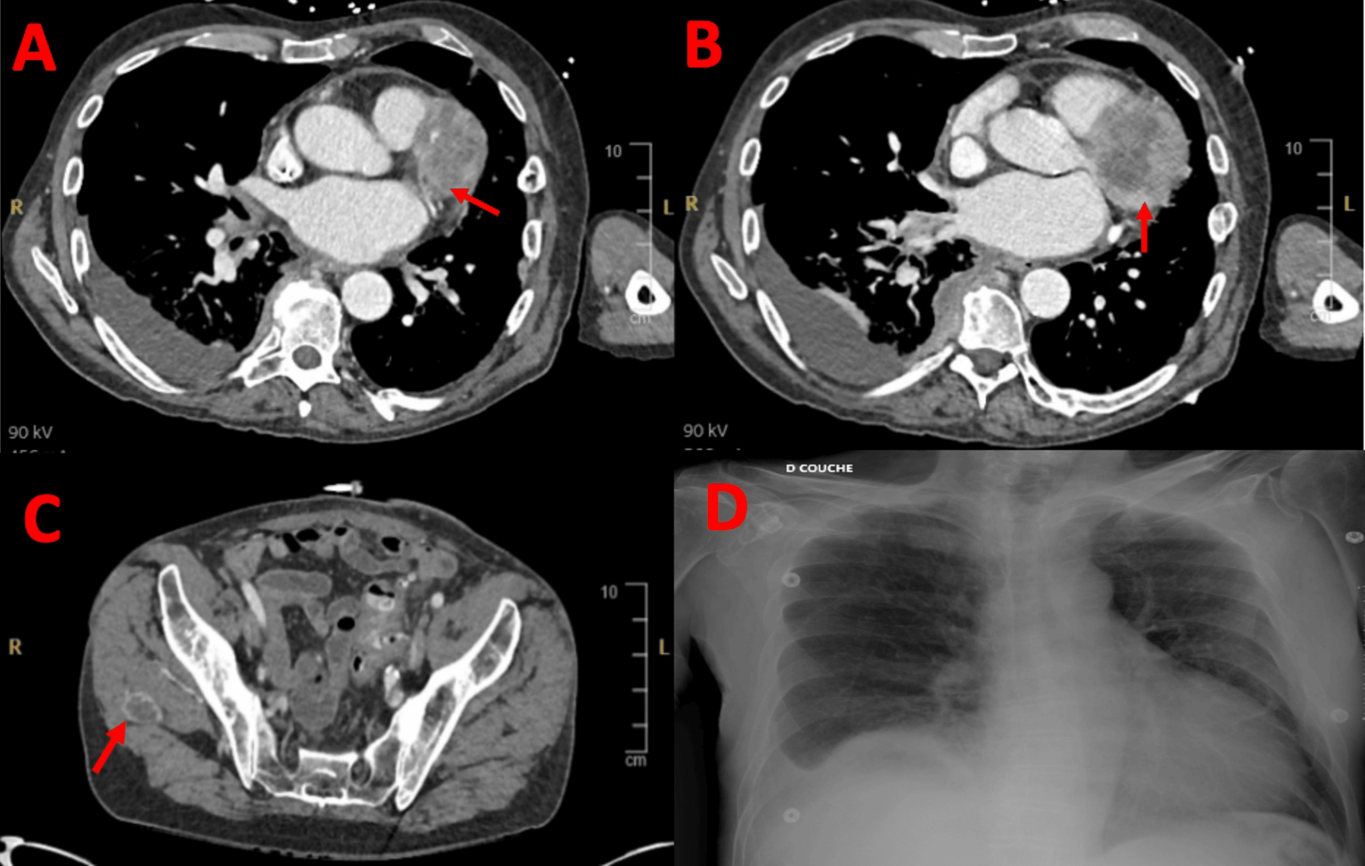
Other imaging modalities. A+B: CT scan images showing the cardiac mass (red arrows). C: CT scan image showing gluteal muscle metastasis. D: Chest X-ray that does not show any specific finding.

A whole-body CT scan performed two days after the cardiac MRI showed the cardiac mass and revealed multiple metastatic lesions involving the liver, kidneys, adrenal glands, peritoneum, gluteal muscle, and lungs, along with evidence of a pulmonary embolism (Figure 5).

Given the patient's history of cancer, the multiplicity of lesions, and the radiological characteristics of the cardiac mass, the diagnosis of cardiac metastasis was retained. Other differential diagnoses of cardiac masses, such as angiosarcoma or fibroma, were considered less likely and subsequently ruled out.

In light of this diagnosis and the poor prognosis, the medical team decided not to proceed with coronary angiography, intravascular imaging, or functional testing, which are typically performed one week after initial angiography in similar cases without a clear etiology. The case was discussed in a multidisciplinary meeting with the oncology team, and a palliative care approach was adopted.

The patient was discharged on oral apixaban 5 mg twice a day, amiodarone 200mg, and bisoprolol 5 mg per day with continuation of previously prescribed medications, including empagliflozin and methotrexate. No additional therapies were initiated.

Due to the severity of the metastatic disease and limited life expectancy, it was also decided not to implant an implantable cardioverter-defibrillator (ICD), to forgo further diagnostic investigations.

The patient was readmitted one month after discharge due to clinical deterioration. During this hospitalization, the patient developed a poorly tolerated episode of ventricular tachycardia. In accordance with the previously agreed-upon palliative approach, the decision was made not to intervene. The ventricular tachycardia subsequently degenerated into ventricular fibrillation, followed by asystole. The patient's death was then pronounced.

## Discussion

Cancer of the oropharynx is one of the most frequently diagnosed cancers worldwide, representing the seventh largest incidence of new cancer in men and fourteenth amongst women. LSCC accounts for approximately 3.0% of oropharyngeal carcinomas and can metastasize to the lungs, heart, and bones, as well as to nearly all organ systems [1]. Although unusual, cardiac involvement from oral cancers has been reported, particularly from lingual squamous cell carcinoma. A systematic review identified 23 cases of cardiac metastases from head and neck cancers, of which 12 originated from the tongue [1]. These rare cases underline the importance of considering cardiac metastasis in patients with a history of oral cancer presenting with cardiac symptoms. Possible explanations for these metastases relate to the hematogenous spread of squamous cell neoplasms through the coronary arteries, direct contiguous extension, and retrograde lymphatic flow [1].

Cardiac metastases may present with chest pain, increasing shortness of breath, hypotension, cardiac tamponade, or arrhythmias. Syncope, while less common, may be an initial presenting symptom. In the review of cardiac metastases from lingual carcinoma, syncope occurred in 16.1% of cases, potentially due to arrhythmias, conduction blocks, or obstructive cardiac masses [1,6]. Ventricular arrhythmias, such as ventricular tachycardia (VT), may occur due to direct myocardial infiltration, fibrosis, or disruption of electrical pathways by the tumor mass [3,4,7].

Electrocardiographic changes are frequent but nonspecific in cardiac metastases. These may include T-wave inversions, ST-segment elevations, and conduction abnormalities. ST-segment elevation mimicking acute coronary syndrome was observed in 63.2% of patients with cardiac metastases from oral cavity cancer [2,5].

Cardiac troponin elevation is a key biomarker in the diagnosis of myocardial infarction (MI), but it may also be observed in non-obstructive conditions such as myocardial infarction with non-obstructive coronary arteries (MINOCA) and cardiac metastases. In the context of MINOCA, troponin levels are consistently elevated and often meet the diagnostic criteria for MI. However, peak values are generally lower than those observed in type 1 MI. Several studies have reported median high-sensitivity troponin concentrations in MINOCA ranging from 200 to 1,000 ng/L, whereas type 1 MI typically presents with levels exceeding 5,000 ng/L [8,9]. In contrast, troponin elevation associated with cardiac metastases is more heterogeneous and non-specific. It may result from direct myocardial infiltration, pericardial involvement with secondary myocardial irritation, or tumor-associated inflammation. In such cases, troponin levels tend to remain modest (often below 500 ng/L) and rarely reach thresholds indicative of extensive myocardial necrosis [2,6,10]. Consequently, interpretation of troponin elevation in these clinical scenarios must be contextualized using multimodal imaging and the overall clinical picture.

Extrinsic compression of coronary arteries by cardiac tumors can mimic acute coronary syndrome, both clinically and on imaging. This phenomenon, though rare, has been documented. For instance, extrinsic compression of the left main coronary artery by a metastatic squamous cell carcinoma was reported, resulting in significant luminal narrowing without underlying atherosclerosis [10]. This mechanism must be considered in patients with known malignancies presenting with ischemic symptoms and angiographic findings that are discordant with typical coronary artery disease.

In our case, the initial clinical presentation, characterized by ventricular tachycardia, repolarization abnormalities on the ECG, and echocardiographic findings, was highly suggestive of an acute coronary syndrome secondary to atherosclerotic disease. However, coronary angiography did not reveal any significant obstructive lesions typically associated with atherosclerosis. Instead, the findings raised the possibility of alternative etiologies, such as an intramural hematoma or, in light of the patient’s oncologic history, extrinsic compression of a coronary artery by a metastatic cardiac mass. It is important to note that conventional coronary angiography has limitations in detecting non-atherosclerotic causes of coronary narrowing, particularly when the mechanism involves external compression or invasion by adjacent pathological processes such as tumors [8].

Imaging is central to the detection and characterization of cardiac metastases. Transthoracic echocardiography is often the first-line modality, allowing visualization of masses, wall motion abnormalities, and pericardial effusions. However, cardiac MRI offers superior tissue characterization, localization, and assessment of tumor infiltration [1]. Indeed, cardiac MRI is considered the most definitive imaging modality for evaluation of myocardial metastasis and delineation of intracardiac tumor thrombi [1]. It can help differentiate between benign and malignant lesions and guide biopsy planning. PET-CT further contributes to detecting metastatic spread and monitoring recurrence, as demonstrated in cases of cardiac metastases from oral carcinoma [1,6].

In our case, a PET-CT scan was not performed, as multiple metastatic lesions were already identified on the whole-body CT scan, and the management strategy was palliative. This case highlights the essential role of cardiac magnetic resonance imaging (MRI) as a complementary diagnostic tool alongside ECG, echocardiography, and coronary angiography in the evaluation of cardiac masses. Whole-body CT imaging also proves valuable, as the identification of additional lesions supports the metastatic origin of the cardiac mass.

Cardiac metastases are typically observed in patients with advanced-stage malignancies and are generally associated with a poor prognosis. As such, management is most often palliative [6,11].

Furthermore, this case underscores the fact that cardiac metastases can occur late, even after a period of apparent oncologic remission. It emphasizes the importance of long-term clinical follow-up in cancer survivors, as cardiac symptoms may occasionally reflect delayed metastatic spread.

## Conclusions

This case underscores the critical importance of considering cardiac metastasis as a potential etiology in patients with a history of cancer who present with atypical arrhythmias or features suggestive of acute coronary syndrome, particularly when coronary angiography fails to reveal significant obstructive disease. The findings highlight the diagnostic limitations of coronary angiography in identifying non-atherosclerotic causes of ischemia, such as extrinsic arterial compression by tumor masses. In contrast, cardiac MRI proved to be a pivotal tool, enabling detailed characterization of myocardial involvement and guiding clinical decision-making. The identification of a cardiac mass in conjunction with widespread metastatic disease shifted the clinical focus toward palliative management, reflecting the grave prognosis typically associated with cardiac metastases. Ultimately, this case reinforces the value of multimodal imaging, especially cardiac MRI, and a high index of suspicion in oncologic patients with unexplained cardiac symptoms. It also illustrates the necessity for a multidisciplinary approach in managing such complex cases, balancing diagnostic clarity with realistic therapeutic goals.
